# Application for monitoring the patients with suicidal tendencies

**DOI:** 10.1192/j.eurpsy.2026.12076

**Published:** 2026-07-31

**Authors:** A. C. Bratan, V. Tau, E. D. Franti, C. Niculae, A. V. Tebeanu, M. Dascalu, A. Ciobanu

**Affiliations:** 1 Research Institute for Artificial Intelligence; 2 University of Medicine and Pharmacy Carol Davila; 3 IMT Bucharest, Bucharest, Romania; 4 AREUS Technology, Boston, United States; 5 National University of Science and Technology Politehnica Bucharest, Bucharest, Romania

## Abstract

**Introduction:**

The application present into this paper was developed during a collaboration with two psychiatric hospitals from Romania, and it is new because it is based on data obtained from patients who attempted suicide and survived against their will, and thus they provide to the doctors many details about what they felt during the moments when they tried to kill themselves. The recordings from the patients’ interviews showed that at the moments when they tried to kill themselves, the patients experienced intense emotions specific to suicide, that remained impregnated for many hours or even days in their voices.

**Objectives:**

The application was developed with a Convolutional Neural Network (CNN) that was trained to identify the specific pattern for each level of intensity of the emotions specific to suicide, imprinted in the voices of three categories of patients:

- first category included 49 patients that just attempted suicide and survive (they were selected only patients with NO mental issues); these patients had strong emotions specific to suicide;
- second category included 31 patients who were treated in the hospital during 4 weeks (after they survived to suicide attempts); these patients had emotions specific to suicide with medium intensity;
- third category included 49 patients with depression and suicidal ideation (meaning that they had only thought about suicide, without implementing any concrete action to kill themself ); these patients had emotions specific to suicide with low intensity.

**Methods:**

The patients were informed about the study objectives, stages, and data protection measures, and were assured of their right to withdraw at any time. All subjects gave informed consent and assent for inclusion before participating in the study. The patients’ interviews were audio recorded using two Sony ICD-PX240 recorders and were saved in MP3 format.

**Results:**

The CNN achieved an **81.6% accuracy** rate in classification, the evolution of the accuracy parameter during the 10 epochs.

**Image 1:**
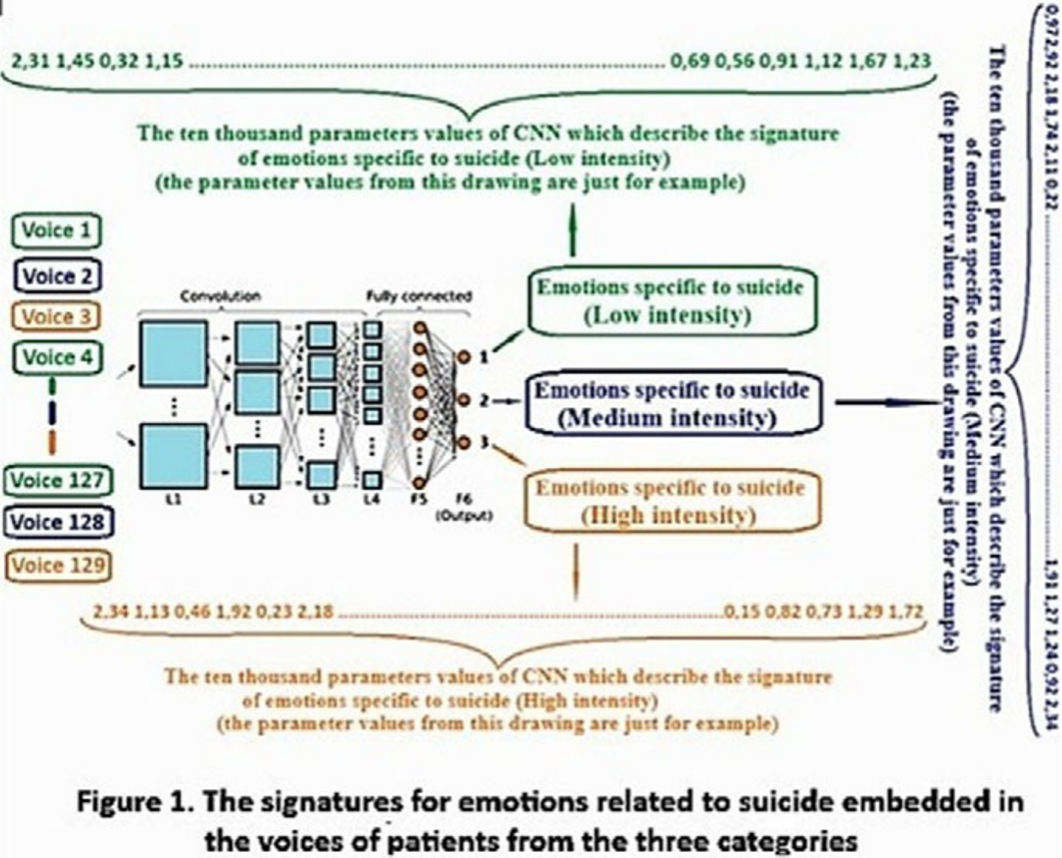
Image 1: long description.

**Conclusions:**

The application helps doctors to identify those patients who continue to have strong suicidal tendencies, after they were treated in the hospital, and they will continue the treatment till their suicidal tendencies will decrease. The accuracy of the application not depends on the language spoken by the patients, because CNN analyses only the emotions impregnated into the patients’ voices. The AI algorithm extracts over ten thousand parameters from the patient’s voice and therefore it can detect the correct level of suicidal tendencies of the patients, including those patients who are lying during the interview about their suicidal tendencies.

**Disclosure of Interest:**

None Declared

## Long descriptions

### Image 1: Long description

The diagram illustrates a data processing pipeline for voice analysis using a Convolutional Neural Network, or C N N.

* Input (Left): A vertical stack of labels representing voice samples, starting from Voice 1, Voice 2, Voice 3, and Voice 4 at the top, followed by a break, and ending with Voice 127, Voice 128, and Voice 129 at the bottom.

* Central Architecture: The voice inputs feed into a C N N diagram consisting of six layers labeled L 1 through L 6. L 1, L 2, and L 3 represent Convolution layers with blue square feature maps. L 4, L 5, and L 6 represent Fully connected layers with dense node-and-line connections. L 6 is the Output layer.

* Classification (Center-Right): Three arrows point from the output layer to three distinct categories:

1. Emotions specific to suicide (Low intensity) in green.

2. Emotions specific to suicide (Medium intensity) in dark blue.

3. Emotions specific to suicide (High intensity) in brown.

* Parameter Signatures (Perimeter): Each intensity category is linked to a bracketed string of numerical values representing a signature.

- The Low intensity signature (Top) includes values like 2,31, 1,45, 0,32, and 1,15.

- The Medium intensity signature (Far Right, vertical) includes values like 0,972, 2,18, and 1,74.

- The High intensity signature (Bottom) includes values like 2,34, 1,13, 0,46, and 1,92.

Text notes indicate these ten thousand parameter values describe the signature of emotions specific to suicide for each intensity level.

Navigate back to Image 1:.

